# Supplementary figures and images for: Lactoferrin and Lysozyme Inhibit the Proteolytic Activity and Cytopathic Effect of Naegleria fowleri Enzymes
Source: Pathogens. 2024 Jan 3;13(1):44. doi: 10.3390/pathogens13010044 (PMC10819050; doi:10.3390/pathogens13010044)

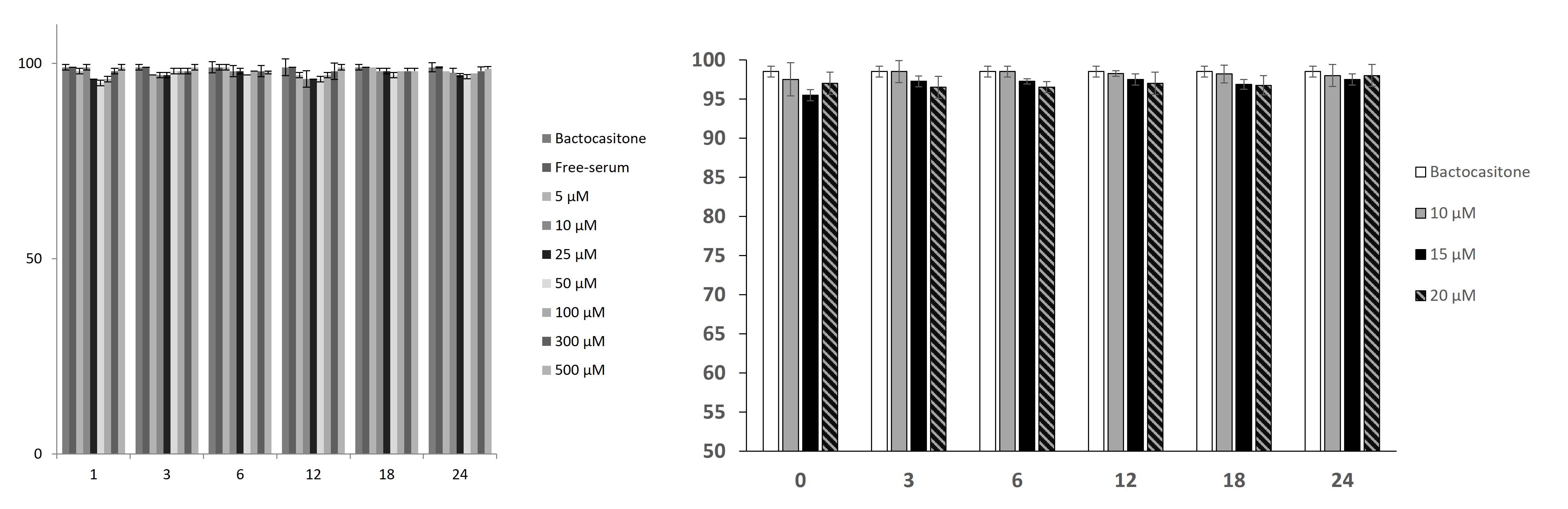

Supplement: Supplementary file 1 [file pathogens-13-00044-s001.zip › Figure S1.jpg]

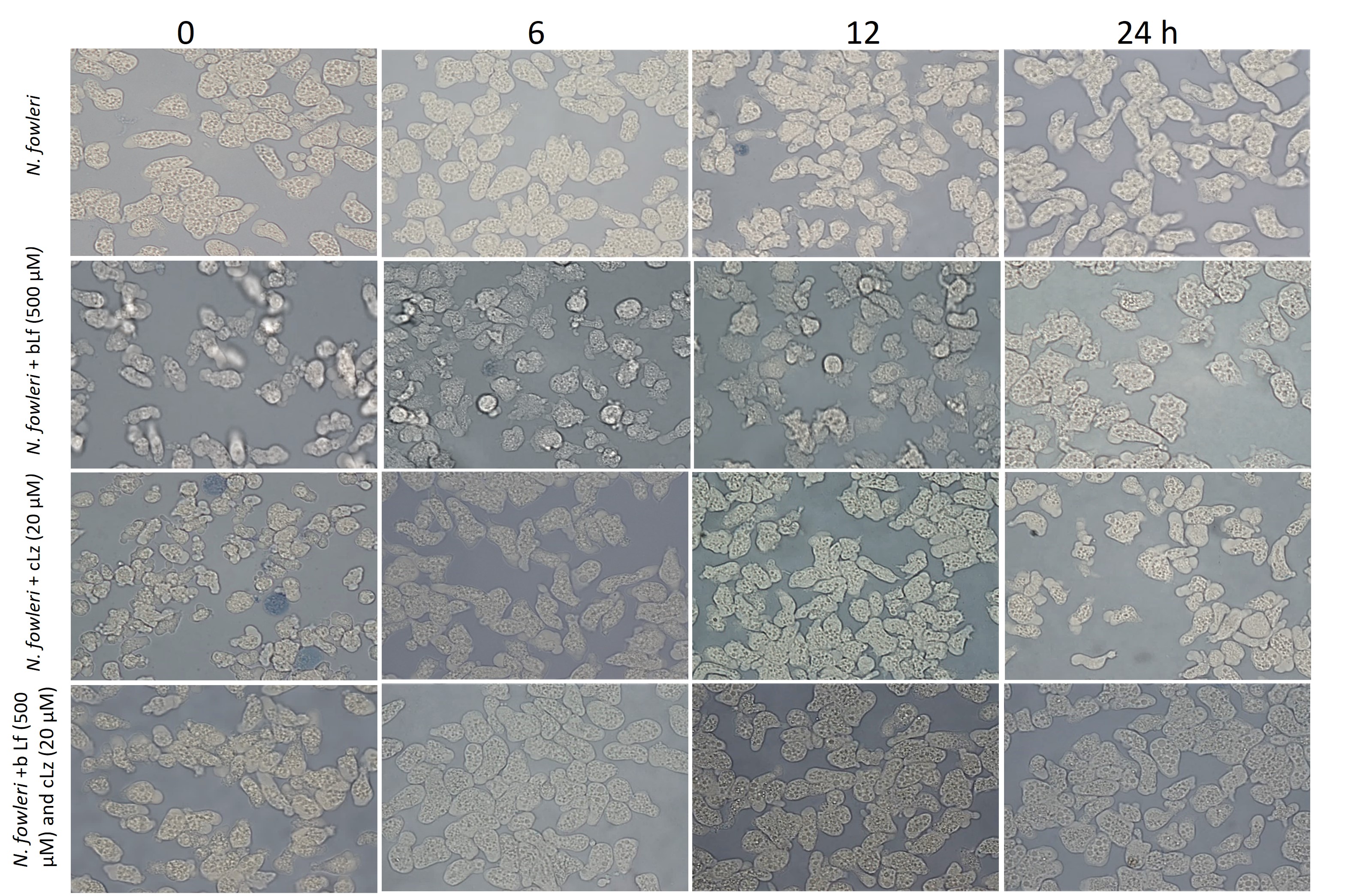

Supplement: Supplementary file 1 [file pathogens-13-00044-s001.zip › Figure S2.jpg]
